# Alterations in Mouse Hypothalamic Adipokine Gene Expression and Leptin Signaling following Chronic Spinal Cord Injury and with Advanced Age

**DOI:** 10.1371/journal.pone.0041073

**Published:** 2012-07-16

**Authors:** Gregory E. Bigford, Valerie C. Bracchi-Ricard, Mark S. Nash, John R. Bethea

**Affiliations:** 1 The Miami Project to Cure Paralysis, University of Miami Miller School of Medicine, Miami, Florida, United States of America; 2 The Department of Neurological Surgery, University of Miami Miller School of Medicine, Miami, Florida, United States of America; 3 The Department of Rehabilitation Medicine, University of Miami Miller School of Medicine, Miami, Florida, United States of America; Oregon Health & Science University, United States of America

## Abstract

Chronic spinal cord injury (SCI) results in an accelerated trajectory of several cardiovascular disease (CVD) risk factors and related aging characteristics, however the molecular mechanisms that are activated have not been explored. Adipokines and leptin signaling are known to play a critical role in neuro-endocrine regulation of energy metabolism, and are now implicated in central inflammatory processes associated with CVD. Here, we examine hypothalamic adipokine gene expression and leptin signaling in response to chronic spinal cord injury and with advanced age. We demonstrate significant changes in fasting-induced adipose factor (FIAF), resistin (Rstn), long-form leptin receptor (LepRb) and suppressor of cytokine-3 (SOCS3) gene expression following chronic SCI and with advanced age. LepRb and Jak2/stat3 signaling is significantly decreased and the leptin signaling inhibitor SOCS3 is significantly elevated with chronic SCI and advanced age. In addition, we investigate endoplasmic reticulum (ER) stress and activation of the uncoupled protein response (UPR) as a biological hallmark of leptin resistance. We observe the activation of the ER stress/UPR proteins IRE1, PERK, and eIF2alpha, demonstrating leptin resistance in chronic SCI and with advanced age. These findings provide evidence for adipokine-mediated inflammatory responses and leptin resistance as contributing to neuro-endocrine dysfunction and CVD risk following SCI and with advanced age. Understanding the underlying mechanisms contributing to SCI and age related CVD may provide insight that will help direct specific therapeutic interventions.

## Introduction

Traumatic spinal cord injury (SCI) initiates a myriad of primary and secondary mechanisms [1], [2] causing neuronal damage and death, sustained neurological deficits, autonomic and immune dysfunction, and significantly high risk of morbidity and mortality [3]. With major advancements in medical practices [4] including operative and non-operative treatment strategies [3], [5] and primary rehabilitation [6], there has been a dramatic increase in the long-term survival rates of persons with SCI [7], [8]. As such, a consequential shift from mortality in SCI related to acute-phase renal, uroseptic, and respiratory complications, to that of chronically acquired all-cause cardiovascular disease (CVD) has become pervasive [6]–[11]. Several risk factors for CVD and related neuro-endocrine/metabolic disorders, described as the *cardiometabolic syndrome*, are prevalent in SCI, and include central obesity [12]–[15], significant dyslipidemia [16]–[22] and depressed plasma HDL-C [16]–[21], [23], [24], as well as impaired fasting glucose and increased prevalence of diabetes mellitus [6], [25]. Moreover, these risk factors are observed earlier in the lifespan and at increased frequencies in SCI [26], and current evidence suggests an accelerated trajectory of aging of body systems [27]–[30] and specifically premature aging of the cardiovascular and neuro-endocrine systems [31]. Although substantial evidence supports that CVD risk factors and related nuro-endocrine/metabolic disorders are prevalent in SCI, the biological mechanisms contributing to these comorbidities have yet to be explored.

It is well understood that adipose-derived peptide hormones, described as adipokines, contribute to both peripheral and central neuro-endocrine regulation of energy metabolism [32], [33], and that dysregulated expression of several of these factors promote pro-inflammatory responses and metabolic dysfunction [34] and are implicated in the pathogenesis of obesity, diabetes mellitus and CVD [35]. Increasing evidence support that several adipokines, including fasting-induced adipose factor (FIAF) and resistin (Rstn) are expressed in various regions in the central nervous system (CNS), and exhibit significant changes in mRNA expression following modeled CNS injury [36], [37]. FIAF and Rstn have been found in key hypothalamic areas responsible for energy balance [37], however, their signaling and physiological effect are poorly understood, and importantly, their role in central inflammatory and metabolic processes with chronic SCI or with advanced age are not defined.

It has been established that the adipokine leptin governs physiological effects on energy homeostasis through hypothalamic pathways mediated by its cognate long form receptor (LepRb) [36], [38]–[42]. LepRb initiates Jak2/Stat3 signaling pathways in subpopulations of neurons in the arcuate nucleus (ARC) of the hypothalamus, activating the transcription of the precursor poly-peptide proopiomelanocortin (POMC) which in turn triggers neuro-endocrine pathways associated with metabolic rate, mobilization of energy stores as well as many other growth related processes [42]–[46]. Importantly, prolonged LepRb activation and Jak2/Stat3 signaling induces suppressor of cytokine signaling 3 (SOCS-3) expression mediating feedback inhibition [47], [48], which is now understood as a mechanism contributing to acquired leptin resistance and subsequent disruption of neuro-endocrine/metabolic function [49], [50].

Hypothalamic inflammation associated with leptin resistance [51]–[54] has been linked in part to endoplasmic reticulum (ER) stress [54], [55], where several (ER) stress transducers have been defined. Phosphorylation of ER transmembrane proteins inositol-requiring protein-1 (IRE1), protein kinase RNA (PKR)-like ER kinase (PERK) and its downstream effector eukaryotic translation initiation factor-2 (eIF2α) contribute to transcriptional activation of a complex signaling network, termed the *uncoupled protein response* (UPR) [56]. Moreover, reduced ER capacity or increased levels of ER stress induce a higher degree of obesity when experimentally challenged with a high fat diet [54], [57], [58]. Although these mechanisms have been implicated in metabolic dysfunction, whether or not dysregulated leptin signaling and acquired leptin resistance are induced by chronic SCI or advanced age, has yet to be explored.

Here we investigate central mechanisms that may contribute to over-arching SCI pathophysiology as it relates to CVD risk and neuro-endocrine/metabolic dysfunction, and explore phenotypic similarities with advanced age. We provide evidence that hypothalamic adipokine gene expression is significantly altered chronically following SCI and with advanced age, as well as significant attenuation of hypothalamic LepRb expression and Jak2/Stat3 signaling. In addition, mobilization of the ER stress response and UPR is observed in both conditions. These findings provide evidence for leptin resistance following chronic SCI and with advanced age, which may contribute to neuro-endocrine/metabolic dysfunction, obesity and CVD risk.

## Materials and Methods

All animal protocols were approved by the University of Miami Institutional Animal Care and Use Committee (IACUC) and are in accordance with National Research Council guidelines for the care and use of laboratory animals. Young (2–4 months) and aged (13–15 months) C57Bl/6 female mice were used in all experiments described.

### Traumatic SCI

Surgeries were performed at the Animal and Surgical Core Facility of the Miami Project to Cure Paralysis according to protocols approved by the IACUC of the University of Miami. Contusion injury was induced with the Infinite Horizon device adapted to the mouse. In brief, mice were anesthetized with an intraperitoneal injection of ketamine (80–100 mg/kg) and xylazine (10 mg/kg). Complete anesthetization was determined by the lack of a stereotypical retraction of the hindpaw in response to a nociceptive stimulus. Mice were then subjected to a laminectomy at vertebrae T9 and the exposed spinal cord was injured at a predetermined impact force of 70 kdynes (severe injury). Sham-operated animals underwent all surgical procedures, including laminectomy, but their spinal cords were not injured. After surgery, animals were housed separately and treated with subcutaneous lactated Ringer's solution to prevent dehydration. Manual bladder expression was performed twice daily. Prophylactic antibiotic gentamicin was administered daily for 7 days to prevent urinary tract infections. Animal tissue was harvested 4-weeks post SCI.

### Total ribonucleic acid (RNA) isolation and Quantitative RT-PCR

Total RNA was isolated from mice hypothalamus using the Qiagen RNAeasy mini kit according to the manufacturer's instructions. Two μg of RNA were reverse transcribed using omniscript reverse transcriptase (Qiagen). Real-time PCR was performed with the Rotor-Gene 3000 Real Time Cycler (Corbett Research) on cDNA samples amplified with TAQurate GREEN Real-Time PCR MasterMix (Epicentre Biotechnologies) and primers for FIAF, Rstn, LepRb and SOCS3 (Table 1). Relative expression was calculated by comparison with a standard curve after normalization to β-actin. Between group differences in mRNA expression levels were analyzed using one-way analysis of variance (ANOVA), followed by Tukey *post hoc* comparison (GraphPad, Prism) and reflect percent change from naïve young (NY) control animals. Single group comparison of sham-operated young and aged animals were analyzed using a two-tailed student's t-test (GraphPad, Prism) and reflect percent change from appropriate naïve control animals. Data are expressed as mean ± SEM. A significance level of p<0.05 was accepted as different from control. n = 5 for each group, and each sample was run in triplicate.

**Table 1:** Gene Primers for Quantitative RT-PCR.

Gene	Genebank #	Primer Pairs	Tm/Product
**LepRb**	NM_146146	*Forward*: 5′- ACTCTGGTCAGCAACGATAAACTA *Reverse*: 5′- GAAAAATGTCTGGGCCTCTGTCTC	53.2°C/150 bp
**FIAF**	AF278699	*Forward*: 5′- GCCACCAATGTTTCCCCCAATG *Reverse*: 5′- TACCAAACCACCAGCCACCAGAGA	57.7°C/118 bp
**Rstn**	NM_022984	*Forward*: 5′- CTTTCATTTCCCCTCCTTTTCCTT *Reverse*: 5′- AGTCTTGTTTGATCTTCTTGTC	50°C/109 bp
**SOCS3**	NM_007707	*Forward*: 5′- TCTTTGCCACCCACGGAACC *Reverse*: 5′- CTCGCCCCCAGAATAGATGTAGTA	57.1°C/108 bp

### Protein extraction and immunoblot analysis

Mice hypothalami were harvested and homogenized in a Dounce homogenizer with extraction/lysis buffer (w/v) (20 mM Tris–HCl, pH: 7.5, 150 mM NaCl, 1% Triton X-100; 1 mM ethylenediaminetetraacetic acid, 1 mM ethylene glycol tetraacetic acid, 2.5 mM pyrophosphate, 1 mM β-glycerophosphate) containing protease and phosphatase inhibitor cocktails (Sigma) and then centrifuged at 15, 300× *g* for 2 minutes. Lysates were mixed with 2x Laemmli loading buffer. Equal amounts of protein were resolved on 10–20% gradient Tris-HCl Criterion pre-casted gels (Bio-Rad, Hercules, CA), to separate proteins with a wide range of molecular weights, transferred to polyvinylidene fluoride (PVDF) membranes and placed in blocking buffer (0.1% Tween-20, 0.4% I-block in PBS) overnight (38). Membranes were then incubated with primary antibodies followed by the appropriate HRP-conjugated secondary antibody. Visualization of the signal was enhanced by chemiluminescence using a Phototope-HRP detection kit (Cell Signaling). Quantification of bands corresponding to changes in protein levels was made using scanned densitometric analysis and NIH Image Program 1.62f, and normalized to β-Actin or Jak2, Stat3, IRE1, PERK, eIF2α, where appropriate. Between group differences in immunoblots were analyzed using one-way analysis of variance (ANOVA), followed by Tukey *post hoc* comparison (GraphPad, Prism) and reflect percent change from naïve young (NY) control animals. Single group comparison of sham-operated young and aged animals were analyzed using a two-tailed student's t-test (GraphPad, Prism) and reflect percent change from appropriate naïve control animals. Data are expressed as mean ± SEM. A significance level of p<0.05 was accepted as different from control. n = 8 for each group, and each sample was run in triplicate.

### Perfusion Fixation

4-weeks post-SCI, animals were anesthetized as described above, then received an intracardial injection of heparin (0.1 cc) and perfused transcardially with physiological saline, followed by 100 ml of 4% paraformaldehyde in phosphate-buffered saline (PBS). The brains were removed and placed in 4% paraformaldehyde at 4°C for overnight, then transferred to 20% sucrose in 0.1 M PBS until sectioned.

### Immunohistochemistry

Animals were perfused with 4% paraformaldehyde solution as described above, and brains were processed for cryostat sectioning (Leica SM 2000R sliding microtome). Serial coronal sections (50 μm) (−0.7 mm to −2.4 mm Bregma) [59] were stored in free-floating cryostat media (30% ethylene glycol, 30% sucrose, 0.1 M PBS, pH 7.4) at −20°C then rinsed with 0.1 M PBS (pH 7.4) Tissue sections were blocked/permeabilized by treatment with 5% normal goat serum (Vector Laboratories Inc., Burlingame, CA, USA) and 0.4% Triton X-100 (Sigma). Sections were incubated for 48 hours at 4°C with either LepRb or NeuN primary antibodies (1∶200). Primary antibody binding was detected with Alexa Fluor secondary antibody conjugates (1∶500, Molecular Probes, Eugene, OR, USA). Controls lacking the primary antibody were run in parallel. Sections were counterstained with DAPI and coverslipped with Vectashield mounting medium (Vector Laboratories Inc., Burlingame, CA, USA) for confocal analysis (Olympus, FluoView 1000, scanning confocal microscope).

### Antibodies

Rabbit polyclonal anti-Leptin Receptor (long-form, 1∶500, Abbiotec), rabbit polyclonal anti-Jak2^P^ (1∶1000, Cell Signaling), rabbit polyclonal anti-Jak2^Total^ (1∶1000, Cell Signaling), rabbit polyclonal anti-Stat3^P^ (1∶1000, Cell Signaling), rabbit polyconal anti-Stat3^Total^ (1∶1000, Cell Signaling), rabbit polyclonal anti-SOCS3 (1∶500, AbCam), mouse monoclonal anti-β-Actin (1∶2000, Cell Signaling), mouse monoclonal anti-PERK^P^ (1∶1000, Cell Signaling), rabbit polyclonal anti-PERK^Total^ (1∶1000, Cell Signaling), rabbit polyclonal anti-IRE1^P^ (1∶1000, AbCam), rabbit polyclonal anti-IRE1^Total^ (1∶1000, AbCam), rabbit polyclonal anti-eIF2α^P^ (1∶1000, Cell Signaling), rabbit polyclonal anti-eIF2α^Total^ (1∶1000, Cell Signaling).

## Results

### Adipokine, LepRb, and SOCS3 gene expression are significantly altered following SCI and with advanced age

**Figure 1:**
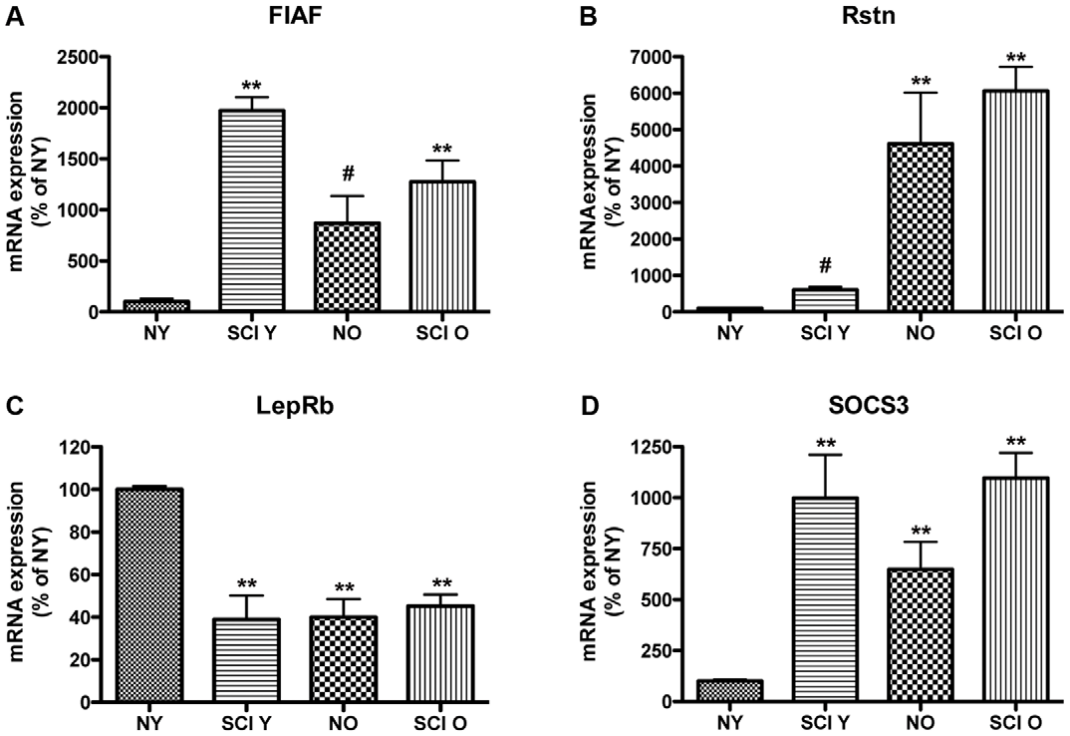
mRNA analysis of FIAF, Rstn, LepRb, and SOCS3 in hypothalamus from naïve young (**NY**)**, SCI young** (**SCIY**)**, naïve aged** (**NO**) **and SCI aged** (**SCIO**) **mice.** Quantification of mRNA expression levels show that FIAF is significantly increased in SCIY, NO and SCI O animals compared to NY control. SCIY and SCIO are also significantly increased when compared to NO (**A**). Rstn mRNA levels are significantly increased in SCIY, NO and SCIO animals compared to NY control. NO and SCIO are also significantly increased when compared to SCIY (**B**). LepRb mRNA levels are significantly reduced in SCIY, NO and SCIO when compared to NY control (**C**). SOCS3 mRNA levels are significantly greater in SCIY, NO and SCIO when compared to NY control (**D**). Statistics are according to data analysis methods described. p≤0.05. n = 5 for each group.

Adipokines modulate central inflammatory responses and metabolic pathways. To investigate whether chronic SCI or advanced age affect central adipokine levels and LepRb signaling intermediates, FIAF, Rstn, LepRb, and SOCS3 gene expression levels were examined using quantitative rt-PCR of hypothalamic extracts from the naïve young (NY), SCI young (SCIY), naïve aged (NO) and SCI aged (SCIO) condition (Figure 1). We observed that FIAF mRNA is significantly increased in the SCI young, naïve aged, and SCI aged animals when compared to naïve young control, and SCI results in significant increases above both the naïve young and naïve aged mice (Figure 1A). Importantly, sham-operated young and aged animals also exhibit significantly increased FIAF mRNA levels when compared to naïve control (Figure S1A), indicating that the differences observed in the SCI condition may not reflect SCI independently. Both naive aged and SCI aged animals show a significant increase in Rstn mRNA levels, when compared to the naïve young control, with no significant difference observed in the SCI young compared to naïve young control (Figure 1B). In addition, the relative amount of LepRb mRNA is significantly reduced in SCI young, naïve aged and SCI aged animals when compared to the naïve young control (Figure 1C), whereas mRNA for the leptin signaling inhibitory intermediate SOCS3 is significantly increased in SCI young, naïve aged and SCI aged animals. (Figure 1D). Sham-operated animals showed no significant difference in Rstn, LepRb, and SOCS3 mRNA level when compared to appropriate naïve control (Figure S1A). These results provide evidence that the hypothalamic adipokine genes and related leptin signaling genes are significantly changed following SCI and with advanced age, which may affect central inflammatory and pathological processes.

### Chronic SCI and advanced age induce a significant decrease in LepRb protein expression, Jak2/Stat3 signaling and concomitant significant increase in SOCS3 protein expression

**Figure 2:**
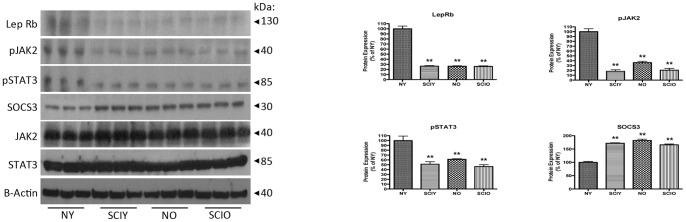
Immunoblot analysis of LepRb and SOCS3 and Jak2/Stat3 signaling in hypothalamus from naïve young (NY), SCI young (SCIY), naïve aged (NO) and SCI aged (SCIO) mice. LepRb expression is significantly decreased in SCIY, NO and SCIO animals when compared to the NY control. Similarly, Jak2 phosphorylation and Stat3 phosphorylation are both attenuated in SCIY, NO and SCIO when compared to the NY control. Conversely, the expression level of SOCS3 is significantly elevated in SCIY, NO and SCIO when compared to the NY control. Jak2^Total^ and Stat3^Total^ were used as internal standards. β-Actin was used as a protein loading control. Statistics are according to data analysis methods described. p≤0.05. n = 8 for each group.

Since LepRB mediates the anorexigenic effect of leptin in the CNS, we next examined whether chronic SCI or advanced age affected hypothalamic LepRb signaling (Figure 2). Consistent with our mRNA data, we observed that LepRb protein expression is significantly reduced in SCI young, naïve aged, and SCI aged animals when compared to naïve young control. Similarly, Jak2 tyrosine phosphorylation and Stat3 tyrosine phosphorylation are also significantly reduced in SCI young, naïve aged, and SCI aged conditions. In contrast, the expression of the leptin signaling inhibitor SOCS3 is significantly increased with SCI young, naïve aged, and SCI aged animals, when compared to naïve young control, also consistent with our mRNA data. Sham-operated animals showed no significant difference in LepRb, Jak2 (phosphorylated), Stat3 (phosphorylated), and SOCS3 protein expression when compared to appropriate naïve control (Figure S1B). These data suggest that hypothalamic leptin signaling through LepRb is significantly reduced with chronic SCI and advanced age, and may contribute to impaired hypothalamic leptin signaling associated with leptin resistance.

### Hypothalamic ER stress and activated UPR following SCI and with advanced age

**Figure 3:**
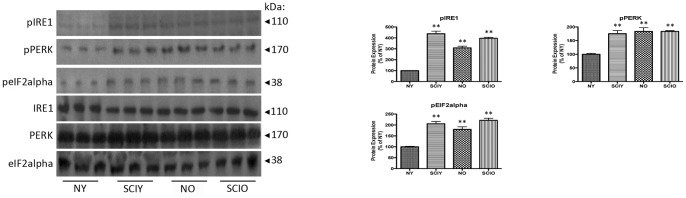
Immunoblot analysis of ER stress and UPR activation in hypothalamus from naïve young (**NY**)**, SCI young** (**SCIY**)**, naïve aged** (**NO**) **and SCI aged** (**SCIO**) **mice.** IRE1 phosphorylation, PERK phosphorylation and eIF2α phosphorylation are each significantly increased in SCIY, NO and SCIO when compared to the NY control. IRE1^Total^, PERK^Total^, and eIF2α^Total^ were used as internal standards. Statistics are according to data analysis methods described. p≤0.05. n = 8 for each group.

ER stress and the UPR have been shown to play a central role in hypothalamic leptin resistance [54]. Therefore, we analyzed the activation of the ER stress response and the UPR in hypothalami of naïve young control, SCI young, naïve aged, and SCI aged animals (Figure 3). The three cellular stress transducer proteins, IRE1, PERK and eIF2α showed significantly increased phosphorylation in SCI young, naïve aged, and SCI aged animals when compared to naïve young control, indicating increased ER stress and an activated UPR. Sham-operated animals showed no significant difference in IRE1 (phosphorylated), PERK (phosphorylated), and eIF2α (phosphorylated) protein expression when compared to appropriate naïve control (Figure S1C). These data show significantly increased ER stress in the hypothalamus, and provide evidence for central leptin resistance following chronic SCI and with advanced age.

### LepRb localize in subpopulations of cells corresponding to the arcuate nucleus (ARC) of the hypothalamus and are significantly reduced following chronic SCI and with advanced age

**Figure 4:**
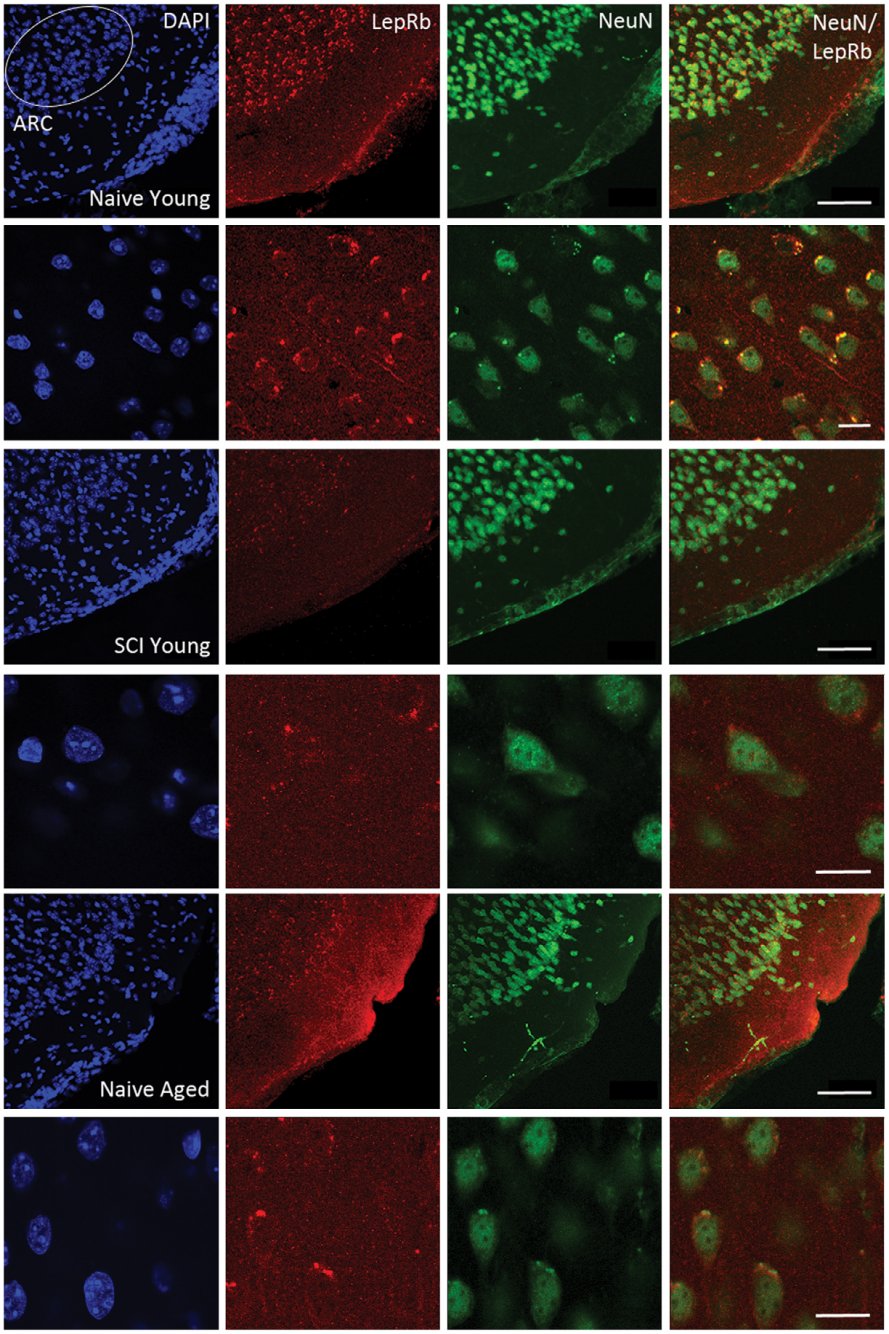
Confocal images of LepRb localization in hypothalamic ARC neurons in naïve young (**NY**)**, SCI young** (**SCIY**) **and naïve aged** (**NO**) **mice.** Mouse brain coronal sections (50 μM; −0.7 mm to −2.4 Bregma) were immunostained with LepRb (Red), the neuronal marker NeuN (Green) and counterstained using DAPI (Blue). In NY mice, brain regions corresponding to hypothalamic ARC (Row 1, Blue) are positively immunostained with LepRb (Row 1, Red) and NeuN (Row 1, Green). Higher magnification (Row 2), confocal images show LepRb (Row 2, Red) immunoreactivity is contained within subpopulations of NeuN (Row 2, green) positive cells and LepRb localizes to peripheral membrane regions on the soma of NeuN positive cells (Row 2, Merged). SCIY mice (Row 3, Row 4) have substantially reduced LepRb (Row 3, 4, Red) immunoreactivity in hypothalamic ARC neurons compared to NY control. Similarly, NO mice (Row 5, 6) display substantially less LepRb (Row 5, Row 6, Red) immunoreactivity in hypothalamic ARC neurons compared to NY control. Scale Bars  = 50 μM (Row 1, 3, 5), 10 μM (Row 2,4,6).

It is well established that the arcuate nucleus (ARC) located within the medio-basal hypothalamus contains subpopulations of leptin responsive neurons [60]–[62] that mediate downstream neuro-endocrine signaling pathways responsible for metabolic control. Confocal images (Figure 4) illustrate the regional distribution and cell type expression of LepRb. Here we show that hypothalamic ARC neurons are positively immunostained with LepRb (red) and the neuronal marker NeuN (green) (Row 1) in the naïve young control. Higher magnification images (Row 2) show that LepRb immunostaining is contained within subpopulations of NeuN positive cells, and that LepRb localizes to somatic peripheral membrane regions. In SCI young (Row 3), ARC neurons had substantially less intense LepRb (red) immunoreactivity, also evident at higher magnification (Row 4). Similarly, in naive aged animals (Row 5), LepRb (red) immunoreacivity was substantially reduced when compared to the naïve young control, and is evident at higher magnification (Row 6). Thus, this supports our molecular and biochemical data showing that LepRb expression is significantly reduced following chronic SCI and with advanced age, and additionally, provides evidence that these changes occur in subpopulations of hypothalamic ARC neurons, known to contribute to neuro-endocrine signaling involved in metabolic control.

## Discussion

Here we show that gene products for the adipokines FIAF, Rstn, and the leptin signaling intermediates LepRb and SOCS3 are present in the mouse hypothalamus, and their expression is significantly altered with either chronic SCI or advanced age. Further, we identified significantly attenuated lepRb protein expression in areas corresponding to the hypothalamic ARC, reduced Jak2/Stat3 signaling, concomitant increases in the leptin signal inhibition protein SOCS3 and activation of the ER stress and UPR proteins IRE1, PERK, and eIF2α. These findings support the idea of adipokine mediated central inflammatory processes and provide evidence for leptin resistance following chronic SCI and with advanced age.

The physical limitations inherent with SCI, as related to movement, musculoskeletal activity and weight bearing contribute to accelerated pathology in cardiovascular and neuro-endocrine health. With these limitations there are subsequent alterations in body composition typified by rapid and long-term declines in muscle mass and increases in central adiposity, which has resulted in the prevalence of obesity with chronic SCI [63], and predictive for cardiometabolic syndrome and CVD [64], [65]. CVD has emerged as the leading cause of mortality in chronic SCI [9], [10], with greater prevalence of several CVD risk factors compared to the able bodied population [9], [10], [66]. Moreover, CVD mortality is significantly greater at earlier ages compared with able-bodied control [10], supporting SCI pathology as mediating an accelerated trajectory of cardiovascular aging [30], [63]. A myriad of physiological changes associated with the neurologic injury and impairment contribute to immediate and long-term effects on body systems [28], [67]. Earlier age related functional declines following chronic SCI have been observed in both the cardiovascular and neuro-endocrine systems. For example, plasma homocysteine [68]–[70] and C-reactive protein (CRP) [71], [72] markers of vascular disease and atherogenesis, are significantly elevated in chronic SCI compared to the able-bodied population, and may contribute to pathological changes in cardiovascular health. Both the extent and neurological level of injury contribute to the development of CVD risk factors in SCI, including dyslipidemia and significant autonomic dysfunction [17], [23], [73], [74]. With this, there is extant imbalance in parasympathetic and sympathetic regulation of cardiovascular control [75] and may involve cardiovascular nuclei within the CNS, which receive neuronal projections from cortical, mesencephalic, as well as hypothalamic regions [76]–[79]. In this regard, the regulation of energy balance and metabolism via specific hypothalamic nuclei, through both neurological innervation and endocrine function, may be greatly affected following SCI. Elevated serum insulin-like growth factor 1(IGF-1) [27], [80], reduced testosterone and human growth hormone [80]–[83] and premature type II Diabetes Mellitus [84] suggest an hastened decline in neuro-endocrine dysfunction. With growing evidence that centrally-derived adipokine gene expression is sensitive to both peripheral and central inflammatory stimuli [85]–[87], it is likely that multiple mechanisms contribute to their signaling dysfunction. Our findings extend previous reports that changes in hypothalamic adipokines may contribute to the activation of signal transduction pathways involved in metabolic dysfunction and consequent CVD with chronic SCI and advanced age.

It has been well established that adipokine signal integration function in metabolic homeostasis, and pathological dysfunction in their gene products, signaling, and coordination are implicated in obesity, diabetes and CVD [35]. FIAF is associated with inflammation of the cardiovascular system, particularly endothelial cells [88] and cardiomyocytes [89]. However, FIAF has also been shown to have beneficial effects on triglyceride lipid metabolism [90], fatty acid oxidation and lipolysis [91] as well as plasma glucose levels and glucose tolerance [92], suggesting an important role in peripheral metabolic homeostasis. Recent evidence has shown FIAF mRNA in the mouse hypothalamus [93] as well as cultured hypothalamic neurons [36], and that hypothalamic FIAF participates in central regulation of energy metabolism through AMP kinase-mediated signaling [88]. Importantly, hypothalamic FIAF gene expression is significantly increased in models of brain injury and inflammation [94], suggesting a signaling role in these pathological processes. Similarly, Rstn has been implicated in a variety of conditions related to the metabolic syndrome, however, the exact mechanism by which Rstn exerts its biological effect are not completely understood. Rstn has been shown to confer glucose intolerance and insulin resistance [95], [96], and although no receptor for Rstn has been identified, induction of SOCS3 intracellularly has been suggested as a potential mechanism by which Rstn inhibits insulin signaling [97]. Additionally, Rstn gene expression levels are significantly upregulated in Apo E −/− mice [98], and are associated with pro-inflammatory markers of atherosclerosis in humans [99], implicating a role in inflammatory processes involved in atherosclerosis. Importantly, Rstn has previously been identified within the murine ARC, colocalizing with POMC neurons, with marked reduction in leptin deficient mice [85], suggesting signaling interaction with leptin in the hypothalamus. Our data show for the first time that chronic SCI and advanced age induces a significant increase in hypothalamic FIAF gene expression. It is important to indicate that sham-operated animals also exhibit significant increases in FIAF, and thus it is remiss to attribute the observed increase in FIAF in the SCI condition solely to SCI pathology. Notwithstanding, the SCI administered is more reflective of the clinical condition, mostly associated with spinal column fracture or dislocation [100], [101], and in this regard, the results reported may in fact represent an important actuality following SCI. Additionally, we observe significantly greater changes following SCI than with advanced age, suggesting that pathological processes involved in SCI have a greater effect on FIAF than processes associated with advanced age. As well, we report for the first time that chronic SCI and advanced age result in significant increases in Rstn gene expression. Interestingly, there is a significantly greater effect with age when compared to SCI, suggesting that processes associated with age have a greater effect on Rstn that pathological processes associated with SCI. Further experiments identifying specific signaling will be necessary to elucidate both physiological function as well as pathological contribution to metabolic dysfunction.

Leptin effects through hypothalamic-mediated pathways are now well characterized, and dysregulation in signaling and subsequent leptin resistance is implicated in chronic inflammation associated with CVD progression. Leptin activates a complex neural network with component orexigenic and anorexigenic signaling, and includes mesolimbic dopaminergic and brainstem integration involved in feeding, satiety and metabolic homeostasis [102], [103]. Both humans and mice with genetic loss of function mutations in either leptin or LepRb manifest severe early onset obesity ([104]–[108], insulin resistance [109], [110], dyslipidemia [111] and other metabolic, neuro-endocrine and immune dysfunctions. Further, evidence has been reviewed [112] indicating that leptin contributes to the pathogenesis of atherosclerotic vascular disease, with positive correlates between plasma leptin levels and arterial distensibility [113], intima-media thickness of the common carotid artery [114], and coronary artery calcification [115]. In fact, in clinically defined coronary atherosclerosis, leptin was determined an independent predictor of future cardiovascular events [116]. As well, leptin has been shown to influence myocardial metabolism and function [117]–[120], and that hypothalamic leptin signaling may normalize myocardial fatty acid oxidation [121]. These reports demonstrate the importance of leptin-mediated signaling in metabolic regulation and overall cardiovascular health. Our data provide evidence that pathological processes involved with chronic SCI and advanced age result in significant dysfunction in hypothalamic leptin signaling, although we do not observe significantly greater leptin signaling deficits in the SCI aged condition when compared to SCI young or naïve aged animals. Reasonably, this may be due to the fact that with advanced age, leptin signaling is substantially hindered that ancillary SCI has insignificant effects. In fact, as the advanced age phenotype represents a progressively and significantly changed metabolic environment, other mechanism may be more prominent with an accompanying SCI. Here we provide evidence of this, illustrating that FIAF gene expression is increased to a greater extent following SCI when compared to advanced age. We also show significantly reduced hypothalamic LepRb gene product and protein expression with chronic SCI and advanced age in areas localized to hypothalamic ARC. Further, our observations of reduced jak2/stat3 in their phosphorylated (or active) state, and increased SOCS3 expression strengthen that LepRb-mediated signaling is attenuated in chronic SCI and with advanced age.

More recent evidence has shown a direct involvement of ER stress and activated UPR in leptin resistance. The maturation and appropriate folding of proteins occurs through the ER luminal system, and is responsive to programs of cell differentiation, environment, and physiological dynamics [56]. Under pathological conditions, the capacity of the ER is exceeded, initiating concurrent signaling cascades, aimed at reducing protein load in the ER, and transcriptionally activating UPR target genes to participate in protein folding and repair. Specifically, the ER trans-membrane proteins IRE1 and PERK, and the signal transducer eIF2α are activated under ER-stress and initiate the transcription of UPR genes [122], [123]. Both *in vitro* and *in vivo* experiments have demonstrated that ER stress and the activated UPR directly effects leptin signaling and induces leptin resistance. For example, cell lines treated with multiple ER stress inducers, significantly inhibited leptin-induced LepRb, jak2 and stat3 phosphorylation [51], [54]. In addition, ER stress has been observed as a prominent feature in the hypothalamus of obese mice, and experimentally induced ER stress and the activated UPR result in leptin resistance in lean mice [54]. Conversely, enhancement of ER capacity augments leptin-stimulated LepRB activation [54], and increases insulin sensitivity and type 2 diabetes in obese mice [124]. These data link obesity, hypothalamic ER stress and the activated UPR, to leptin signaling dysfunction and ultimate leptin resistance, and in this manner, provide biological evidence for leptin resistance in these conditions. We show the induction of ER stress and the activated UPR with chronic SCI and advanced age, although we do not observe significantly greater ER stress and activated UPR in the SCI aged condition when compared to the SCI young and naïve aged condition. In a similar manner to our findings regarding leptin signaling, ER stress and the activated UPR associated with advanced age may reflect a threshold such that accompanying SCI may not incite a significantly greater effect.

We demonstrate the novel findings that chronic SCI and advanced age induce modifications in hypothalamic adipokine genes, dysfunction in LepRb mediated signaling, increased ER stress and activation of the UPR, providing evidence for leptin resistance, which may contribute to metabolic dysfunction and CVD risk in these conditions. Developing an understanding of centrally derived adipokine signaling will help elucidate their physiological role in inflammatory processes, as well as define their contribution to dysfunction under pathological conditions. In particular, leptin signaling involves many central and peripheral processes and tissues, and further experiments will be necessary to define phenotypic changes with chronic SCI and advanced age, and may provide insight into leptin mediated mechanisms involved in metabolic dysfunction and CVD risk and the development of appropriately directed therapeutic countermeasures

## Supporting Information

Figure S1 **Naïve and sham-operated young and aged analysis of hypothalamic adipokine mRNA, leptin signaling intermediates, ER stress and UPR activation.** Quantification of mRNA expression levels show that FIAF is significantly increased in sham-operated young (SY) compared to naïve young (NY) control and in sham-operated aged (SO) compared to naïve aged (NO) control (**A**). Rstn, LepRb and SOCS3 mRNA expression levels are not significantly different in sham-operated young and aged animals when compared to appropriate control (**A**). LepRb expression, Jak2/Stat3 phosphorylation, and SOCS3 expression are not significantly different in sham-operated young and aged animals when compared to appropriate naïve control (**B**). IRE1, PERK, and eIF2α phosphorylation is not significantly different in sham-operated young and aged animals when compared to appropriate control (**C**). Jak2^Total^, Stat3^Total^, IRE1^Total^, PERK^Total^, and eIF2α^Total^ were used as internal standards. β-Actin was used as a protein loading control. Statistics are according to data analysis methods described. p≤0.05. n = 5 for each group.
(TIF)
