# Supplementary figures and images for: Alterations in Mouse Hypothalamic Adipokine Gene Expression and Leptin Signaling following Chronic Spinal Cord Injury and with Advanced Age
Source: PLoS One. 2012 Jul 16;7(7):e41073. doi: 10.1371/journal.pone.0041073 (PMC3397960; doi:10.1371/journal.pone.0041073)

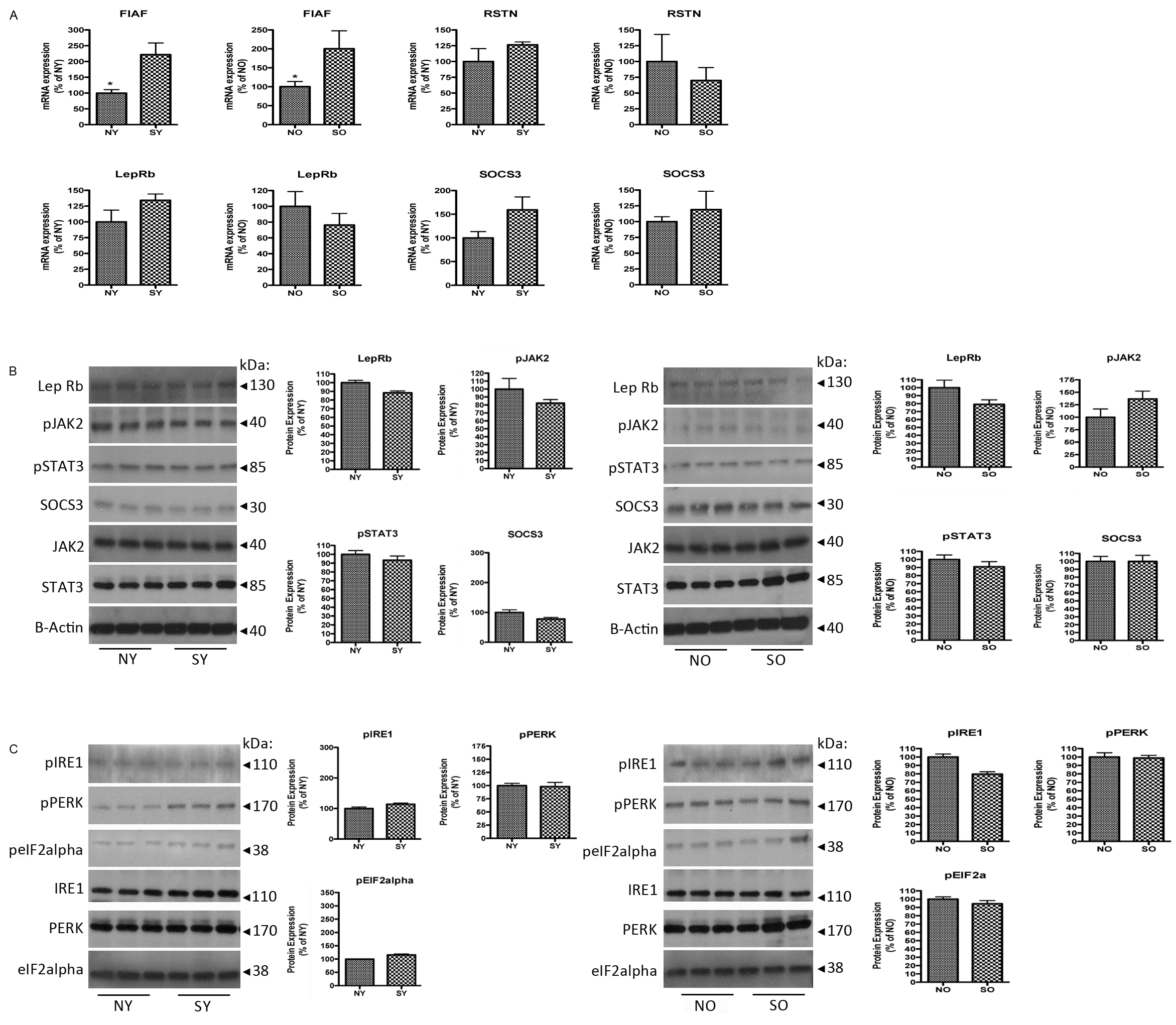

Supplement: Figure S1 — Naïve and sham-operated young and aged analysis of hypothalamic adipokine mRNA, leptin signaling intermediates, ER stress and UPR activation. Quantification of mRNA expression levels show that FIAF is significantly increased in sham-operated young (SY) compared to naïve young (NY) control and in sham-operated aged (SO) compared to naïve aged (NO) control (A). Rstn, LepRb and SOCS3 mRNA expression levels are not significantly different in sham-operated young and aged animals when compared to appropriate control (A). LepRb expression, Jak2/Stat3 phosphorylation, and SOCS3 expression are not significantly different in sham-operated young and aged animals when compared to appropriate naïve control (B). IRE1, PERK, and eIF2α phosphorylation is not significantly different in sham-operated young and aged animals when compared to appropriate control (C). Jak2Total, Stat3Total, IRE1Total, PERKTotal, and eIF2αTotal were used as internal standards. β-Actin was used as a protein loading control. Statistics are according to data analysis methods described. p≤0.05. n = 5 for each group. (TIF) [file pone.0041073.s001.tif]
